# Neonatal sepsis outbreak at the first maternity hospital of Ulaanbaatar

**DOI:** 10.1186/1753-6561-5-S6-P96

**Published:** 2011-06-29

**Authors:** T Muugulug, A Bat-Erdene, Ariungerel Bat-Erdene

**Affiliations:** 1 Dept. for Research and Surveillance of Hai, National Center for Communicable Diseases of Mongoli, Ulaanbaatar, Mongolia

## Introduction / objectives

In-depth analysis for Neonatal sepsis outbreak at the first maternity hospital.

## Methods

We conducted screening by clinical signs among the newborns and then collected clinical samplings from the suspected cases with environmental samplings. Bacteriological analysis was done for all the samples and agents were detected along with antibiotic resistance analysis.

## Results

In Januaryof 2010, 21 neonatal infection cases with 2 death reported were screened by clinical signs. 76.2% of them had cesarean delivery and 23.8% were given normal birth. Clinical presentations were fever 100%, cyanosis 76%, breath distress 65%, abdominal filling 59%, abdominal vein unroll 53%, hyperbilirubinemia 53%, irritability 47% and rigidity 12%. Clinical samplings gathered from 18 neonates and 12 /66.6%/ of them were detected *multi resistant Klebsiella pneumonia.*

there are collected 33 environmental swabs. 27.2% of them have culture positive, including staphylococcus with resistance to ampicillin, streptococcus with sensitive to vancomycin, gram positive and gram negative bacteria and micrococcus resistance to vancomycin. 25% of 8 swabs collected from mothers of 2nd ward had culture positive, including enterococcus with resistance to vancomycin and bacillus cereus.

In air bacteriological sampling results determined 6 staphylococcus hemolyticus. 8 health care workers had Staphylococcus aureus in nasal swabs, 1 had streptococcus pneumonia and *klebsiella pmeumonea* which were detected from 5 neonates nasal swabs, blood and wounds.

## Conclusion

The agents of the outbreak were **Klebsiell?pneumonia** and **staphylococcus** and transmission occured from person to person due to poor performanve of the relevant precautions. The increased workload of the staff, poor condition of the hospital building and sewage system were the supporting reasons of the outbreak.

## Disclosure of interest

None declared.

